# Early Angiographic Resolution of Cerebral Vasospasm with High Dose Intravenous Milrinone Therapy

**DOI:** 10.1155/2015/164597

**Published:** 2015-09-17

**Authors:** F. A. Zeiler, J. Silvaggio

**Affiliations:** Section of Neurosurgery, Department of Surgery, University of Manitoba, Winnipeg, MB, Canada R3A 1R9

## Abstract

*Background*. Treatment of symptomatic delayed cerebral ischemia (DCI) after subarachnoid hemorrhage (SAH) is difficult. Recent studies suggest intravenous (IV) high dose milrinone as a potential therapy. The timing to angiographic response with this is unclear. *Methods*. We reviewed the chart of one patient admitted for SAH who developed symptomatic DCI and was treated with high dose IV milrinone. *Results*. A 66-year-old female was admitted with a Hunt and Hess clinical grade 4, World Federation of Neurological Surgeons (WFNS) clinical grade 4, and SAH secondary to a left anterior choroidal artery aneurysm which was clipped. After bleed day 6, the patient developed symptomatic DCI. We planned for angioplasty of the proximal segments. We administered high dose IV milrinone bolus followed by continuous infusion which led to clinical improvement prior to angiography. The angiogram performed 1.5 hours after milrinone administration displayed resolution of the CT angiogram and MRI based cerebral vasospasm such that further intra-arterial therapy was aborted. She completed 6 days of continuous IV milrinone therapy, was transferred to the ward, and subsequently rehabilitated. *Conclusions*. High dose IV milrinone therapy for symptomatic DCI after SAH can lead to rapid neurological improvement with dramatic early angiographic improvement of cerebral vasospasm.

## 1. Introduction

The treatment of symptomatic delayed cerebral ischemia (DCI) after aneurysmal subarachnoid hemorrhage (SAH) currently focuses on hypertensive therapy with the potential use of intra-arterial calcium channel or phosphodiesterase inhibitor (PDEI) based vasodilators [1–4]. Angioplasty is also an option for proximal large to medium vessel vasospasm resistant to medical therapy [5–7].

Recently a report has surfaced utilizing high dose intravenous (IV) PDEI based therapy with milrinone [8]. This report documented rapid neurological improvement of symptomatic DCI. Angiographic improvement has also been described with this protocol within 24 hours of administration [8]. It is unknown, despite the early symptomatic improvement, how rapid the angiographic improvement occurs with IV milrinone therapy.

Within this report, we describe a case of symptomatic DCI after SAH treated with high dose IV milrinone therapy, in isolation, leading to rapid symptomatic improvement, and early angiographic near resolution within 1.5 hours of the initiation of therapy. This is to the author's knowledge the earliest documented angiographic response of cerebral vasospasm to high dose IV milrinone therapy.

## 2. Case Presentation

A 66-year-old female, with a past medical history for hypertension, presented to hospital with a 12-hour history of an acute onset severe headache and neck pain. Prior to arrival at hospital, she had progressive deterioration in her level of consciousness (LOC). Upon arrival to the emergency department, she was with GCS of 9 (motor = 5, eyes = 2, and verbal = 2). There was no witnessed seizure activity, and the patient was hemodynamically stable throughout transport. No episodes of hypotension or hypoxemia were recorded. She had a definitive airway obtained via endotracheal intubation and was sent for computed tomography (CT) of the brain.

**Figure 1:**
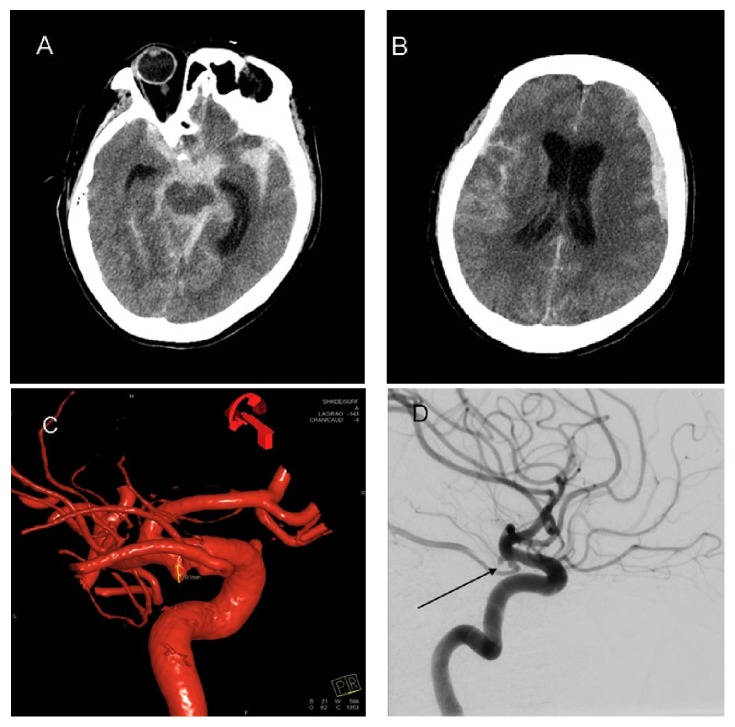
Admission CT and DSA. CT = computed tomography and DSA = digital subtraction angiogram. (A) Axial uninfused CT of the brain at the level of the mesencephalon displaying thick subarachnoid clot in the basal cisterns. (B) Axial uninfused CT of the brain at the level of the lateral ventricles displaying hydrocephalus, a small left acute subdural hematoma and diffuse SAH. (C) Lateral projection of left internal carotid artery injection via DSA displaying a small anterior choroidal artery aneurysm (black arrow). (D) Three-dimensional reconstruction of DSA displaying anterior choroidal artery aneurysm on the inferior portion of the supraclinoid internal carotid artery.

The CT of the brain displayed a Fisher grade 4 SAH with significant clot burden in the interpeduncular, carotid, and ambient cisterns bilaterally (Figures 1(A) and 1(B)). In addition, the imaging displayed hydrocephalus. Computed tomographic angiography (CTA) of the circle of Willis (COW) was obtained and failed to display a discrete source for the SAH. Three-dimensional reconstructions of the CTA were unclear but displayed a questionable small aneurysm in the area of the left anterior choroidal artery.

Given the Fisher CT grade 4, Hunt and Hess clinical grade 4 (with a GCS of 9 and stuporous), and the World Federation of Neurological Surgeons (WFNS) clinical grade 4 SAH with the presence of hydrocephalus, a right frontal external ventricular drain (EVD) was placed. The EVD was left open at 20 cm above the tragus. The patient was transferred to the intensive care unit (ICU) with the plan for a 4-vessel digital subtraction angiogram (DSA), in order to better delineate the suspected anterior choroidal artery aneurysm. The patient improved to a GCS of 9T (motor = 6, eyes = 3, and verbal = T) after EVD placement.

The DSA images confirmed the presence of the aneurysm (Figures 1(C) and 1(D)), and the patient was taken to the operating room for microsurgical clipping 6 hours after admission to hospital.

Intraoperatively the procedure went without complication, with the exception of the need to sacrifice one large sylvian vein. Postoperative CT of brain displayed small subfrontal hypodensity consistent with venous infarct. The patient was clinically unaffected by this.

Postoperatively the patient returned to the ICU where she remained ventilator dependent on pressure support of 20 with a positive end expiratory pressure (PEEP) of 8 cm H_2_O and a fractional inspired oxygen (FiO_2_) requirement of 50%. Chest X-ray displayed pulmonary edema. She had mild tropinemia, with no significant electrocardiogram (ECG) changes. A transthoracic echocardiogram displayed biventricular failure (right worse than left) and global hypokinesis, with an ejection fraction (EF) of 40%, all consistent with catecholamine related subendocardial ischemia. She remained on minimal sedation for comfort during her ICU stay, with a fentanyl infusion at 25 mcg/hr. This was not changed at any point during her ICU admission. Nimodipine was administered only once, at a dose of 60 mg, and resulted in a decrease in MABP from 85 mm Hg to 55 mm Hg. Given this response to nimodipine and the echo results, it was elected to not administer any further doses.

Over the following 5 days after bleed, she remained ventilator dependent secondary to her pulmonary edema. We consistently recorded central venous pressure (CVP) between 12 and 15 mm Hg. Mean arterial blood pressure (MABP) was maintained spontaneously between 80 and 90 mm Hg.

**Figure 2:**
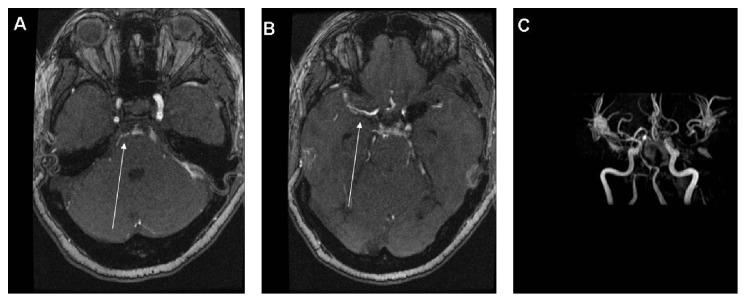
Postbleed day 5 MRI with TOF angiography. MRI = magnetic resonance imaging and TOF = time of flight. (A) Axial TOF sequence at the level of the pons displaying severe basilar artery spasm (white arrow). (B) Axial TOF sequence at the level of the mesencephalon displaying severe right middle cerebral artery vasospasm (white arrow). (C) Three-dimensional TOF reconstruction of the circle of Willis displaying severe spasm of the basilar artery trunk and the middle cerebral arteries bilaterally. The vasospasm is severe enough to prevent TOF signal from identifying the distal basilar and the left proximal middle cerebral artery.

On postbleed day 5, she had a brief episode of right arm weakness lasting for 30 seconds, with return to baseline. There was no documented loss of consciousness. She remained able to obey commands both during and after this brief event. Uninfused CT of the brain failed to display any new abnormality. Electroencephalogram was conducted, with no seizure activity noted. Subsequently magnetic resonance imaging (MRI) of the brain was completed displaying signs of moderate to severe angiographic vasospasm of the basilar trunk, bilateral supraclinoid internal carotid arteries (ICA), and bilateral M1 segments of the middle cerebral artery (MCA) via time of flight (TOF) imaging (Figure 2). Given only the presence of angiographic cerebral vasospasm via TOF MRI in the absence of clinical manifestations, it was elected to monitor the patient clinically.

**Figure 3:**
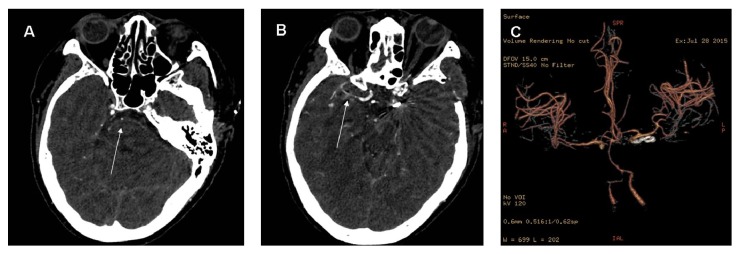
Postbleed day 6 CTA of the COW. CTA = computed tomographic angiography and COW = circle of Willis. (A) Axial CTA image at the level of the pons displaying severe spasm of the basilar artery (white arrow). (B) Axial CTA image at the level of the mesencephalon displaying severe spasm of the right proximal middle cerebral artery (white arrow). (C) Three-dimensional reconstruction of the CTA displaying the COW. Of note is the severe spasm of the basilar artery trunk and the right middle cerebral artery and bilateral proximal anterior cerebral arteries.

On postbleed day 6, the patient became drowsy and unresponsive in the early afternoon. No changes in her hemodynamics were noted during this time, with MABP recorded between 80 and 90 mm Hg. An urgent CTA of the COW was ordered confirming the results from the MRI-TOF obtained the previous evening (Figure 3). Given the significant proximal vasospasm, it was elected to arrange for angioplasty while the patient was optimized medically for the presumed diagnosis of symptomatic DCI secondary to cerebral vasospasm.

In the presence of significant subendocardial ischemia and pulmonary edema requiring ventilatory support, it was elected to avoid hypertensive therapy and trial of a high dose PDEI based therapy with IV milrinone following the previously described protocol from the Montreal Neurological Institute [8]. A 5 mg IV milrinone load over 10 minutes was given (0.1 mg/kg load; weight 55 kg), followed by continuous IV infusion of 0.75 mcg/kg/min. No hypotension or arrhythmia was encountered. The MABP during and after load was maintained at 80 to 95 mm Hg spontaneously.

Within 30 minutes of the IV milrinone load, the patient began to open her eyes spontaneously. Within 40 minutes of the infusion, the patient began to intermittently follow commands with all extremities. The patient was subsequently taken to the angiography suite for planned angioplasty approximately 1.5 hours after IV milrinone bolus.

**Figure 4:**
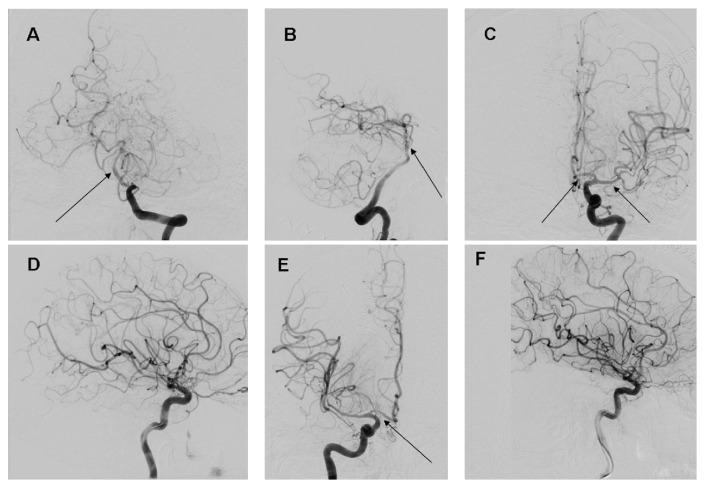
DSA performed 1.5 hours after milrinone administration. DSA = digital subtraction angiogram. (A) AP projection of a left vertebral artery injection displaying good opacification of the basilar artery trunk (black arrow). (B) Lateral projection of left vertebral artery injection displaying good opacification of the basilar artery trunk. (C) AP projection of a left internal carotid artery injection displaying resolution of all left sided vasospasm and normal appearing proximal anterior and middle cerebral arteries (black arrows). (D) Lateral projection of left internal carotid artery injection displaying no distal MCA vasospasm. (E) AP projection of right internal carotid artery injection displaying improvement in proximal middle cerebral artery spasm, with mild residual stenosis (black arrow). (F) Lateral projection of a right internal cerebral artery injection displaying no distal small vessel vasospasm.

The DSA prior to the planned angioplasty displayed significant angiographic resolution of the vasospasm in the basilar and bilateral ICA/MCA territories. Some mild residual spasm was noted at the basilar apex and the right proximal middle cerebral artery (Figure 4). Given the improvement and lack of obvious target of angioplasty, the procedure was aborted and the patient returned to the ICU. It was noted at this time that the patient was back to baseline neurologically.

The patient was maintained on 0.75 mcg/kg/min of IV milrinone for 72 hours, after which we tapered the infusion by 0.25 mcg/kg/min every 24 hours. No complications of the milrinone therapy were encountered. The MABP was maintained spontaneously between 85 and 95 mm Hg while being on the IV milrinone therapy. We were also subsequently able to liberate the patient from the ventilator 2 days after the attempted angioplasty procedure.

Continued improvement occurred during the following 1.5-week ward stay, with an eventual transfer to our stroke rehabilitation unit for ongoing therapy.

## 3. Discussion

A few important points can be gathered from our case. First, IV based PDEI therapy with milrinone led to significant cerebral vasodilation of the proximal large vessel territories. Second, utilizing a high dose IV bolus of milrinone, followed by a continuous infusion, appears to have a dramatic early effect on the angiographic appearance of cerebral vasospasm in our case. With the DSA occurring within approximately 1.5 hours of the IV milrinone load, it can be seen that the effect on the cerebral vasculature is rapid. To our knowledge, this is the earliest documented DSA response to high dose IV milrinone therapy currently in the literature. Third, the symptoms of DCI resolved quite rapidly with the administration of IV milrinone and appeared to be sustained. This mirrors the effects previously described [8]. Fourth, there were no recorded side effects of the milrinone therapy during both bolus and infusion. This also mirrors the previously described reports of milrinone therapy for DCI after SAH [8]. Fifth, our particular patient had a constellation of complications for which milrinone was an ideal solution. The presence of subendocardial ischemia, with biventricular failure, and pulmonary edema requiring ventilation all improved dramatically after milrinone administration. This allowed us to liberate her from the ventilator and improved our ability to follow her neurological exam. Finally, we were able to avoid the potential complications of high dose vasopressor usage that are seen during hypertensive therapy for DCI.

Despite the interesting effects seen in our case, there are significant limitations that need acknowledgment. First, this is a single case that in no way proves that this therapy can or should be utilized as a first line treatment for symptomatic DCI after SAH. The current standard therapy is hypertensive management and should remain so until further prospective study of milrinone therapy has been conducted. Second, the positive results seen in this case cannot be extrapolated to all cases of DCI after SAH given that this is only a single case. The results however are mirrored in previously reported experiences with the described milrinone protocol [8]. Third, we compared premilrinone MRI-TOF and CTA to postmilrinone DSA. Given that these imaging modalities are very different, it is hard to say with 100% certainty that the angiographic response seen during DSA was in fact as dramatic as described. The premilrinone CTA and MRI-TOF may have overrepresented the degree of cerebral vasospasm. However, given the degree of spasm seen on the CTA and MRI-TOF, we would expect the DSA to have displayed at least some significant degree of vasospasm if the milrinone had not been effective. Fourth, one could argue that the clinical effect seen is not a cerebral vasodilatory effect but merely a cardiac index driven effect of the milrinone in the setting of subendocardial ischemia. Unfortunately we did not have a Swan-Ganz catheter or noninvasive biotransmittance cardiac monitor in order to make comments on the cardiac index both pre- and postmilrinone administration. However, the MABP remained completely unchanged during the pre- and postmilrinone administration phases of this patient's care. Thus, an improved cardiac index leading to improved CBF, in the absence of changes in the MABP, as the sole reason for neurological improvement is hard to argue. Similarly, the angiographic response seen cannot be justified with this argument. Fifth, despite no complications in our case, these high doses of milrinone should be utilized with caution. The risk for substantial hypotension and arrhythmia is very real. Finally, there currently is no data to suggest that milrinone based vasodilatory therapy in the setting of DCI after SAH has any impact on patient outcome, despite our positive outcome in this case.

Despite all of the limitations, we believe this case provides an interesting example of the early angiographic and clinical impact that high dose IV milrinone therapy can have in the setting of symptomatic DCI after SAH. At this point in time, this therapy is not standard of care in this setting and requires significant evaluation in a prospective fashion prior to widespread implementation. Future prospective study should include comparison to hypertensive therapy in a randomized controlled fashion as a frontline therapy for symptomatic DCI after SAH with the outcome measures including angiographic response, clinical response, radiological outcome (i.e., presence of strokes during follow-up), and the impact of therapy on clinical outcome.

## 4. Conclusions

High dose IV milrinone therapy for symptomatic DCI after SAH can lead to rapid neurological improvement with dramatic early angiographic improvement of cerebral vasospasm. Further prospective study of this therapy is requirement prior to widespread implementation in this setting.
